# Supplementary material for: Effects of Magnetic Minerals Exposure and Microbial Responses in Surface Sediment across the Bohai Sea
Source: Microorganisms. 2021 Dec 21;10(1):6. doi: 10.3390/microorganisms10010006 (PMC8778929; doi:10.3390/microorganisms10010006)
Supplement: Supplementary file 1 [file microorganisms-10-00006-s001.zip › microorganisms-1490878-supplementary.pdf]

Supplementary Materials for

Effects of magnetic minerals exposure and microbial responses in surface sediment across Bohai Sea

Lei Chen, Mingpeng Wang, Liangyue Chi, Yuntao Li, Weitao Shang, Jianhui Tang, Zhaojie Zhang, Fanghua Liu

*Corresponding author. Email: qsdwmp2018@qfnu.edu.cn (M. Wang) and fhliu@soil.gd.cn (F. Liu)

This file includes:

Supplementary Text
Figures S1 to S6
Tables S1 to S3

Supplementary Text

Figure S1.

The rarefaction curve of 24 samples.

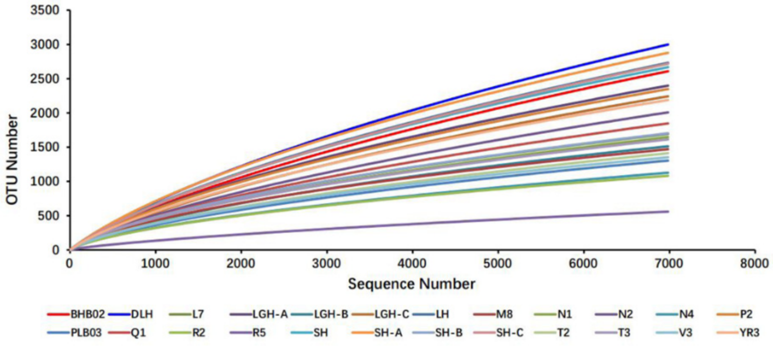

Figure S2.

The relative abundances of the top 10 most abundant bacterial phyla in relation to sediment salinity. Only significant correlations are shown ($p < 0.05$). The strength of each relationship given is based on the linear regression equation.

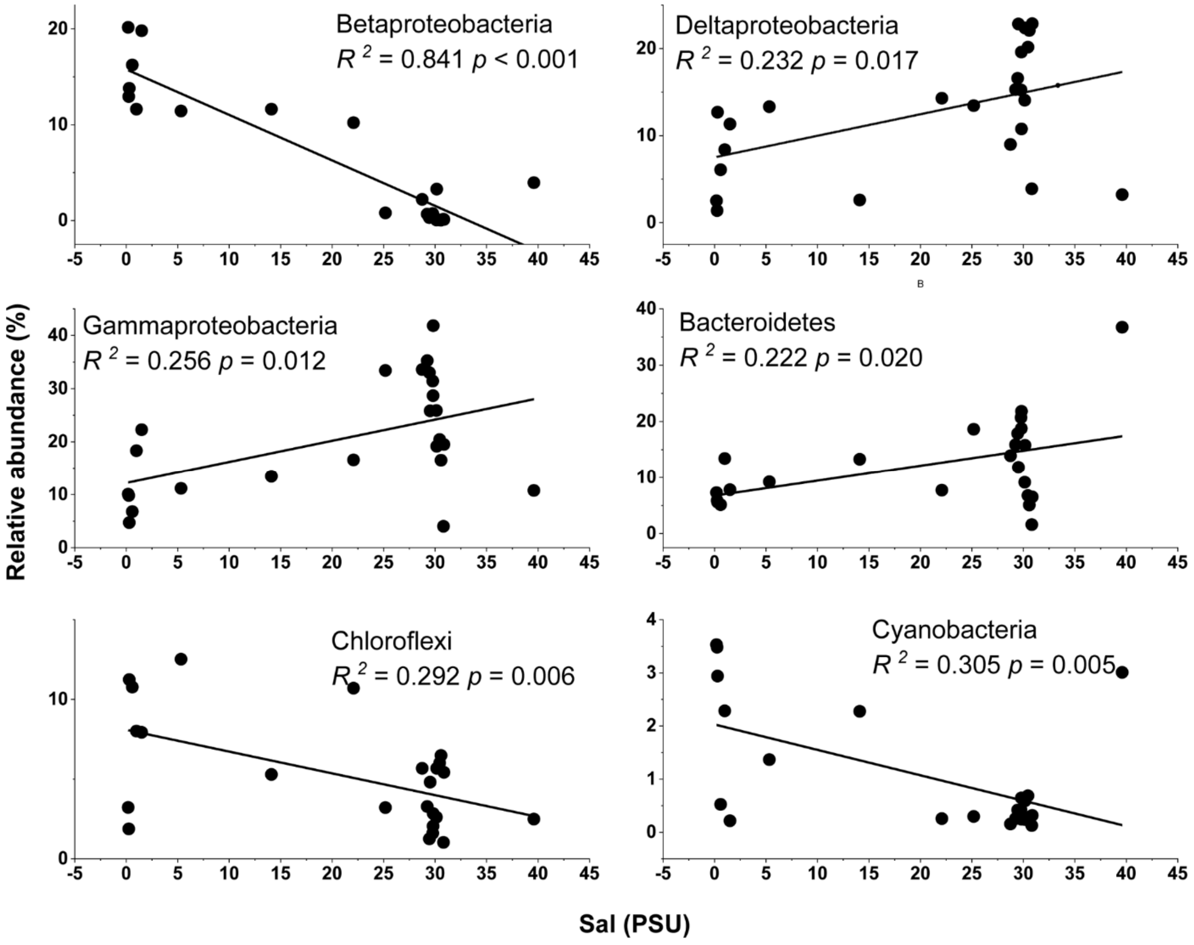

Figure S3.

The relative abundances of the top 10 most abundant bacterial phyla/classes in relation to water pH and sediment DO. Only significant correlations are shown ($p < 0.05$). The strength of each relationship given is based on the linear regression equation. Solid lines show regression lines.

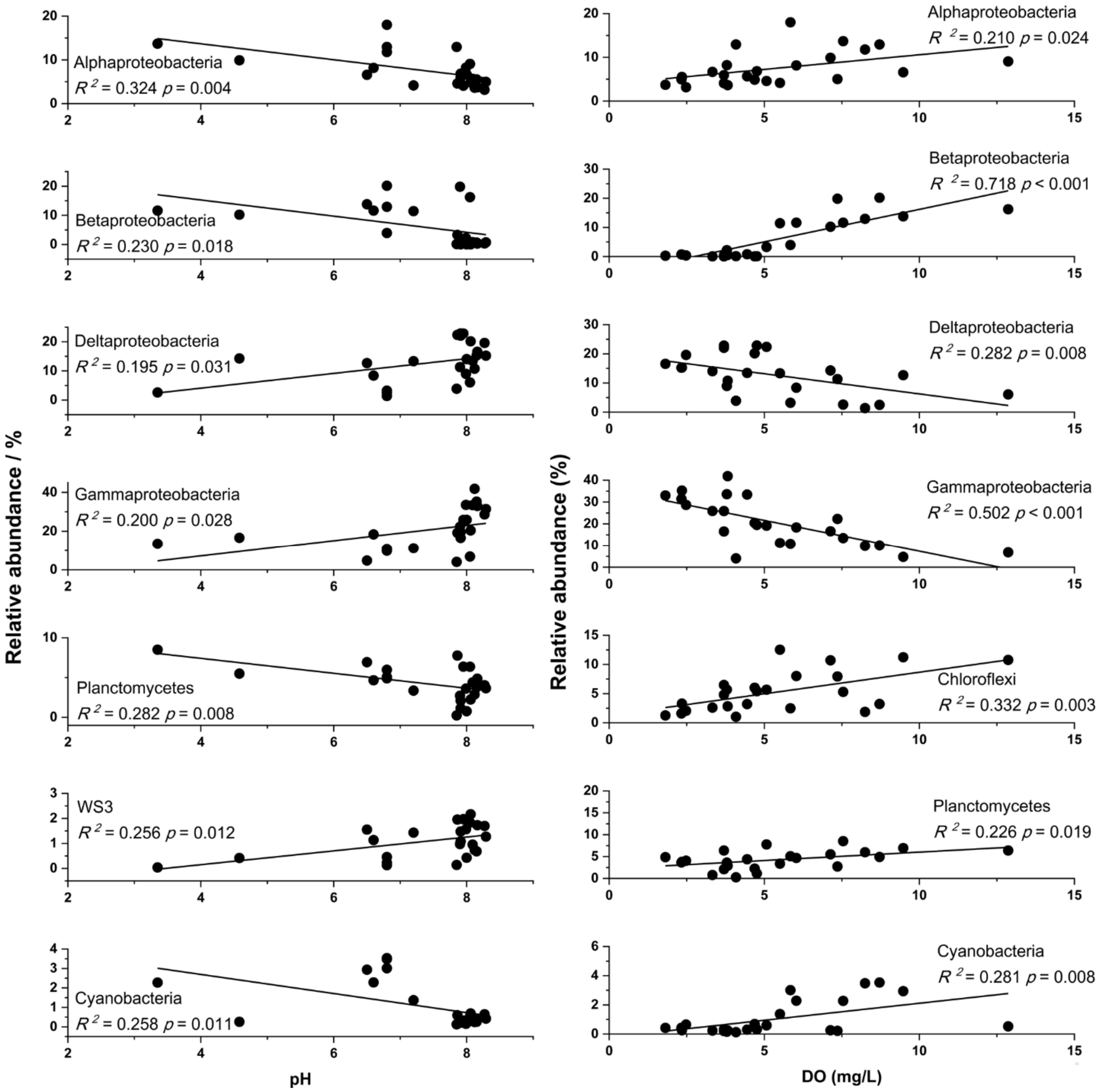

Figure S4.

The relative abundances of top 10 bacterial phyla in relation to sediment Fe content. Only significant correlations are shown ($p < 0.05$).

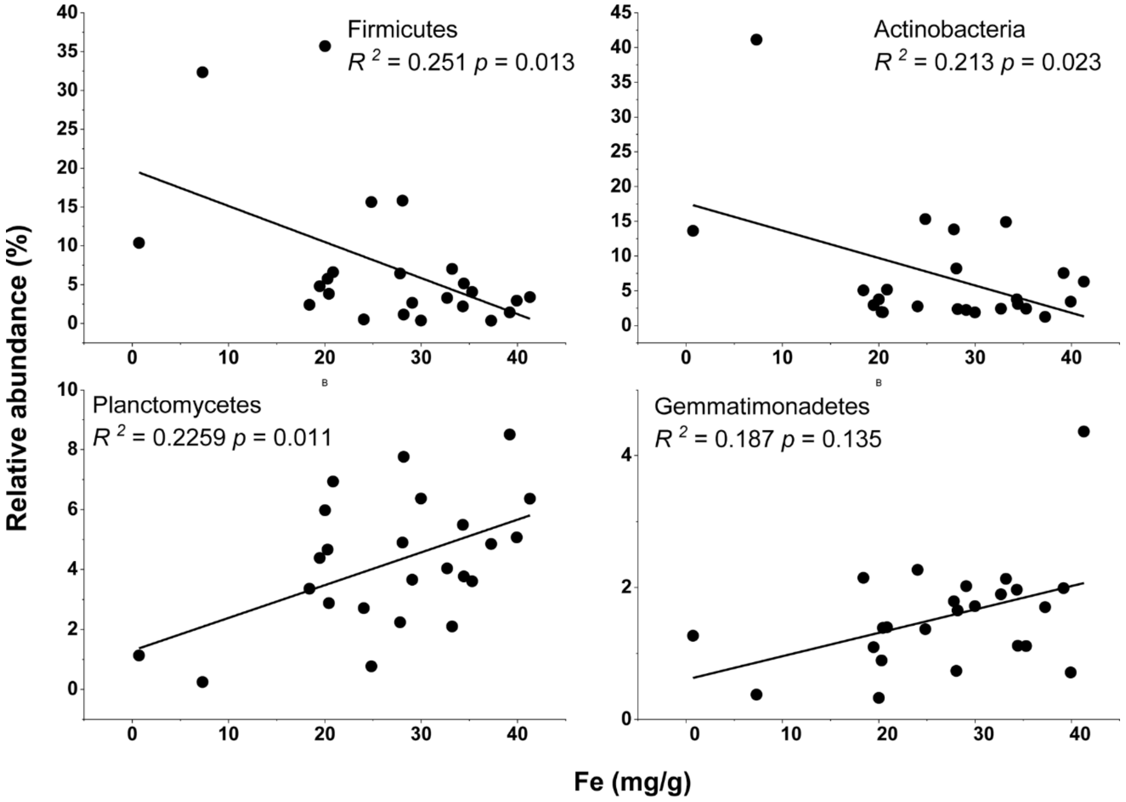

Figure S5.
The relative abundances of the top 10 most abundant bacterial phyla/classes in relation to sediment χlf. Only significant correlations are shown ($p < 0.05$). The strength of each relationship given is based on the linear regression equation. Solid lines show regression lines.

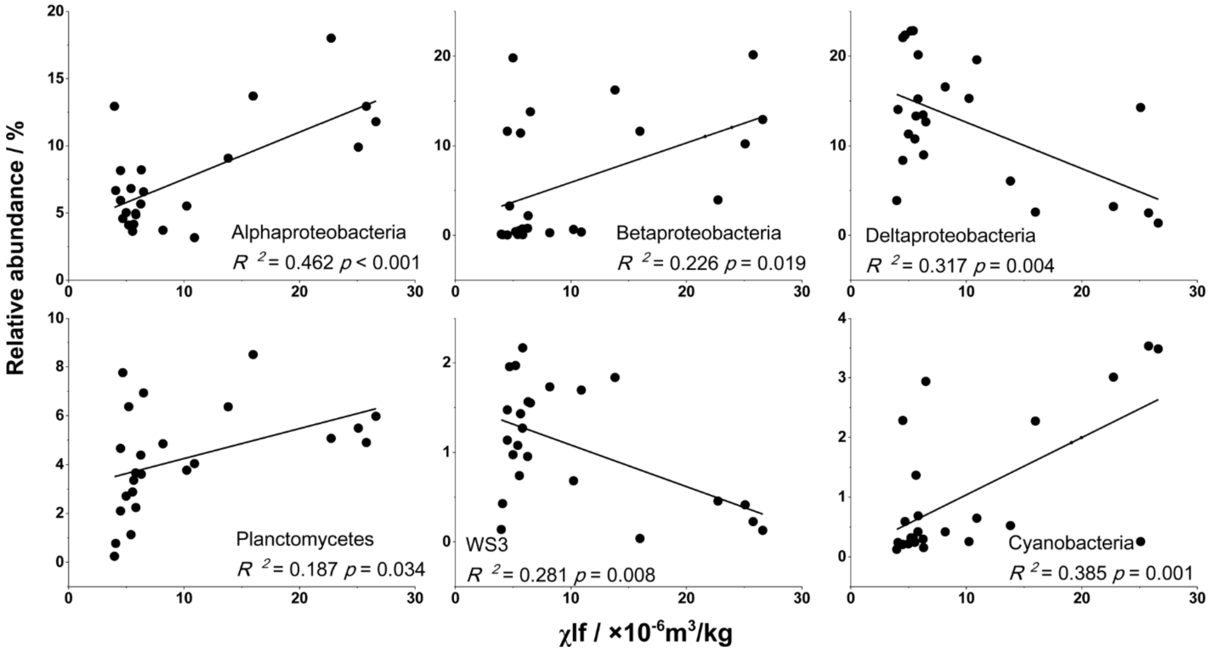

Figure S6.

The relative abundances of the top 10 bacterial bacterial phyla/classes in relation to sediment TOC. Only significant correlations are shown ($p < 0.05$).

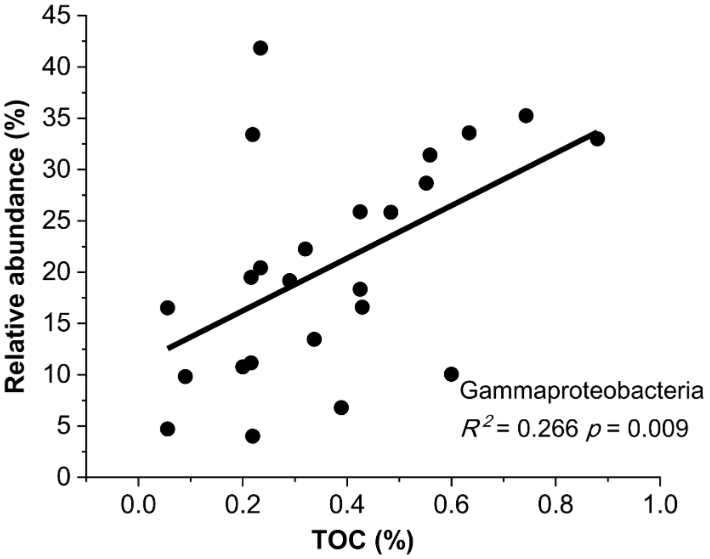

Table S1.

Selected characteristics of the sediments from geographically different sites used in this study.

Sediment	Salinity (PSU) [1]	pH [1]	DO (mg/L) [1]	Fe (mg/g) [2]	χlf [3] (×$10^{-6}m^3/kg$)	TOC (%)[4]	TN (%)[4]
DLH	5.33	7.20	5.51	18.41	5.65	0.216	0.008
LH	0.99	6.60	6.03	20.29	4.50	0.425	0.014
T3	25.18	8.09	4.45	19.47	6.26	0.219	0.013
T2	29.82	8.12	3.82	20.43	5.55	0.234	0.014
SH	0.30	6.50	9.48	20.86	6.50	0.056	0.010
N1	29.24	8.15	2.34	34.43	10.23	0.743	0.075
N2	29.45	8.16	1.81	37.27	8.18	0.880	0.072
LGH-A	0.19	6.80	8.72	28.06	25.78	0.600	0.027
LGH-B	0.25	6.80	8.25	20.02	26.61	0.090	0.010
LGH-C	39.60	6.80	5.84	39.93	22.74	0.200	0.012
Q1	29.80	8.27	2.48	32.69	10.91	0.552	0.061
M8	29.77	8.29	2.33	29.08	5.81	0.559	0.035
SH-A	0.59	8.05	12.86	41.29	13.82	0.389	0.014
SH-B	14.10	3.35	7.55	39.19	15.98	0.337	0.015
SH-C	22.09	4.58	7.13	34.33	25.09	0.429	0.013
L7	30.17	7.86	5.07	28.19	4.70	0.290	0.011
YR3	1.50	7.90	7.36	24.04	4.99	0.320	0.104
BHB02	28.75	7.99	3.80	35.30	6.30	0.634	0.825
P2	29.52	7.95	3.70	29.98	5.21	0.484	0.613
N4	30.86	7.91	4.76	0.72	5.41	0.216	0.050
R2	30.14	8.00	3.33	24.83	4.09	0.425	0.016
R5	30.82	7.85	4.09	7.31	3.98	0.219	0.016
V3	30.45	8.06	4.69	27.81	5.83	0.234	0.014
PLB3	30.58	7.91	3.70	33.211	4.50	0.056	0.032

[1] One measurement by CTD. DO (Dissolved Oxygen) and pH were measured in the water nearest the sea floor.
[2] One measurement by ICP-MS.
[3] One measurement and calculation using a magnetic susceptibility meter.
[4] One measurement by elemental analyzer. TOC: Total Organic Carbon.TN: Total Nitrogen.

Table S2.

Bacterial α-diversity in sediment samples.

Groups	Sample grouping	Chao 1 index	Observed-OTU richness (S)	Shannon Wiener index (H')
Sediments [1]	DLH	7786	2999	7.3
	LH	7098	2732	7.2
	T3	3807	1614	6.2
	T2	3465	1412	5.7
	SH	6424	2668	7.1
	N1	3829	1647	6.2
	N2	5336	2008	6.4
	LGH-A	6387	2399	6.9
	LGH-B	3250	1513	5.6
	LGH-C	5796	2243	6.1
	Q1	4412	1847	6.2
	M8	3095	1470	6.0
	SH-A	6741	2878	7.3
	SH-B	3894	1703	6.2
	SH-C	6774	2719	7.1
	L7	3756	1693	6.3
	YR3	5242	2189	6.6
	BHB02	6733	2609	7.0
	P2	6202	2349	6.8
	N4	3074	1127	5.1
	R2	2344	1182	5.1
	R5	1488	559	2.7
	V3	3034	1353	5.5
	PLB03	2824	1304	5.4

[1] Diversity indices are calculated using a random selection of 6,983 sequences per sediment sample.

Table S3.
Mantel tests showing the Spearman's rank correlations between environmental variables and geodistance.

Variables	ρ	*P*
Salinity	0.121	0.060
Fe	0.079	0.149
χlf	-0.017	0.542
TOC (%)	-0.014	0.549
TN	0.072	0.174
**pH**	**0.191**	**0.021**[1]
DO	0.009	0.411

[1] *P*-value in bold indicates significant difference ($P < 0.05$) tested by Spearman's rank correlation analysis. ENV: environmental variables.
